# Optimizing Management Strategies for Vocal Cord Nodules: A Systematic Review

**DOI:** 10.7759/cureus.75916

**Published:** 2024-12-17

**Authors:** Mohammed H Baali, Mohammad H Shaheen, Meshal F Khan, Abdulaziz A Neazy, Mohammed A Basyuni, Abdulaziz Altowairqi

**Affiliations:** 1 Otolaryngology - Head and Neck Surgery Department, King Abdulaziz Specialist Hospital, Taif, SAU; 2 Otolaryngology - Head and Neck Surgery Department, Hera General Hospital, Makkah, SAU; 3 Otolaryngology - Head and Neck Surgery Department, Al-Noor Specialist Hospital, Makkah, SAU; 4 Otolaryngology - Head and Neck Surgery Department, Maternity and Children's Hospital, Makkah, SAU; 5 Otorhinolaryngology - Head and Neck Surgery Department, King Abdulaziz Specialist Hospital, Taif, SAU

**Keywords:** speech, surgery, vocal cord immobility, vocal cord nodules, voice

## Abstract

Vocal cord nodules (VCNs) can be treated with a variety of therapeutic approaches, with controversy regarding the optimal management. This review provides an overview of the most commonly used management strategies and their outcomes to enhance decision making. We conducted a systematic literature search on PubMed, Web of Science, and Scopus to include relevant original articles published in peer-reviewed journals from inception through April 2024. We used a broad search strategy including the following search keywords: "Vocal" AND "Nodules" OR "Lesions." All interventions were included, such as speech or voice therapy, medication, surgery, and other therapies. Two authors independently conducted initial screening, full-text screening, inclusion, and data collection. Disagreements were settled by consensus or, if persistent, by a senior author. A total of 18 articles were included in the current scoping review. Voice therapy is highlighted as the preferred non-invasive treatment; however, it requires a longer duration to show results. In children, voice therapy is further limited by non-compliance, which may result in delayed recovery and increased psychological and economic burden. Surgery offers immediate improvements but necessitates postoperative voice therapy to prevent recurrence. In hard or mature nodules, surgery showed superior efficacy compared to voice therapy. Pharmacotherapy is principally indicated to treat an underlying disease and is often prescribed as an adjunct to voice therapy. Patient age, duration of the disorder, morphological features of VCNs, and compliance with voice therapy are significant prognostic factors in determining the success or failure of different treatment modalities. We propose a management algorithm that incorporates these key factors to assist in effective management and decision making. In addition, the management of VCNs requires a methodical approach that considers the prognostic factors associated with each treatment modality. We propose a key-factor-based algorithm that incorporates these factors to assist in therapeutic decision making and guide future research toward establishing definitive guidelines.

## Introduction and background

Vocal cord nodules (VCNs), which are non-cancerous growths on the vocal cords, cause symptoms such as voice alterations, hoarseness, and discomfort, including shooting pain or neck pain [1,2]. VCNs are particularly common in children, accounting for 3-26% of vocal disorders and 38-78% of hoarseness cases [3,4]. Voice abuse or overuse is a frequent underlying mechanism in children [4,5], causing the vocal folds to rub together and form callus-like growths [6]. VCNs negatively impact children's physical health and quality of life, potentially leading to symptoms of depression or anxiety [7].

VCNs can be treated with various therapeutic approaches, including surgery and pharmaceutical treatments [8]. Surgical treatment is more challenging in younger patients than in adults. Therefore, a conservative approach combining medical therapy and behavioral therapy, i.e., voice hygiene, is often preferred for children with VCNs [9,10]. One of the causes of poor treatment response in youth is a lack of self-control [10]. Pharmacological interventions like budesonide inhalation can reduce discomfort and inflammation, improving VCN symptoms [11]. However, long-term use may have adverse effects, particularly on a child's growth, which limits the efficacy of such treatment [11]. Conversely, voice therapy has gained attention in recent years due to its success in treating VCNs in children [12,13].

Although voice therapy is the primary treatment for VCNs, little research has described the methods, number of sessions, or length of treatment [14]. Nonetheless, the lengthy treatment and the need to modify voice habits can discourage patients and affect adherence. In such cases, surgery may become the only option. Microsurgery for pediatric VCNs is only performed in exceptional cases, such as questionable diagnoses, total non-response to voice therapy, or a significant worsening of symptoms [15,16]. Due to the diverse and controversial data on the optimal management of VCNs, we conducted this review to provide an overview of the most commonly used management strategies and evaluate their outcomes to identify the most effective approach.

## Review

Methods

Data Sources and Searches

We conducted literature searches in PubMed, Web of Science, and Scopus to identify relevant publications for our scoping review. The search strategy used was as follows: "Vocal" AND "Nodules" OR "Lesions," covering publications from inception through April 2024. Duplicates were removed using EndNote [17], and the final articles were screened using Rayyan [18].

Eligibility Criteria and Screening

Eligibility criteria included primary original publications in peer-reviewed journals examining therapeutic approaches for VCN patients, regardless of age. We included studies covering all forms of interventions, such as speech or voice therapy, medication, surgery, and other therapies. We excluded reviews, case reports, and publications with different objectives. Two authors independently screened titles and abstracts, resolving disagreements by consensus or, if necessary, by consulting a senior author. Full-text screening followed to assess eligibility based on the predefined criteria.

Data Extraction and Synthesis

We extracted data providing an overview of each study, including the country of origin, design, sample size, gender, age, age group, treatment mode, treatment mechanism, evaluated results, follow-up duration, study goal, and key findings. This was completed with a qualitative synthesis of the study aims, treatment approaches and outcomes, and the main findings of the studies.

Results

Studies Flowchart

**Figure 1:**
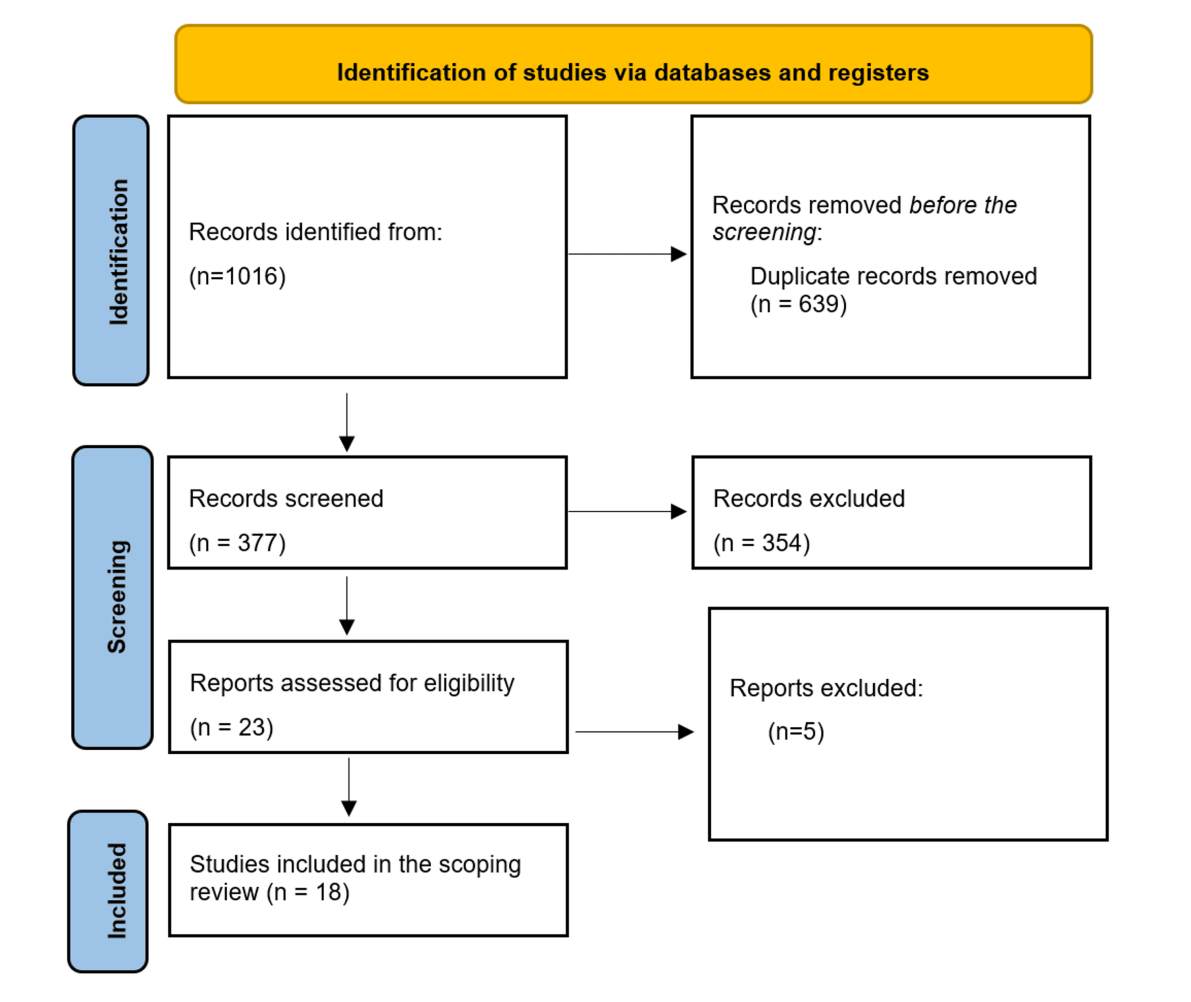
PRISMA flow diagram of database searching and screening. PRISMA: Preferred Reporting Items for Systematic Reviews and Meta-Analyses

The initial database search yielded 1,016 articles. After removing duplicates, 377 articles underwent title and abstract screening. Of the latter, 23 articles were eligible for full-text screening, and 18 met the eligibility criteria for our scoping review (Figure 1) [19-36].

The Main Characteristics of the Included Studies

Five of the included studies were conducted in the United States, three in China, three in India, two in Brazil, and one each in Bangladesh, Indonesia, France, Turkey, and Mexico. Regarding the study design, the majority of the included studies (14 studies) were cohort studies, while four were RCTs, with a total sample size of 1,243 patients. The mean age ranged from five years to 84 years, with 10 of the included articles being conducted on children, seven on adults, and one study on both adults and children (Table 1).

**Table 1:** Summary and baseline characteristics of the included articles. RCT: randomized controlled trial

Studies	Country	Design	Sample size	Gender (male/female)	Age mean (SD)	Adults/children
Liu et al. (2024) [21]	USA	Cohort	368	224/144	5.0 (0.7)	Children
Ma et al. (2021) [24]	China	RCT	17	9/8	7.5 (1.1)	Children
Hseu et al. (2024) [25]	USA	Cohort	23	17/6	2.4-9.9	Children
Martins et al. (2020) [20]	Brazil	Cohort	78	55/23	9.69 (2.36)	Children
Itigi et al. (2022) [26]	India	Cohort	100	65/35	10-84	Adults and children
Palash et al. (2022) [27]	Bangladesh	Cohort	30	12/18	33.53 (7.72)	Adults
Akbulut et al. (2016) [28]	USA	Cohort	19	8/11	40	Adults
Liu et al. (2022) [22]	China	Cohort	194	140/54	5.3 (2.14)	Children
Hartnick et al. (2018) [29]	USA	RCT	112	83/29	8.10	Children
Bakat et al. (2014) [30]	India	Cohort	18	4/14	31.56	Adults
Mobarsa et al. (2019) [31]	India	Cohort	30	22/8	38.73	Adults
Muslih et al. (2019) [32]	Indonesia	Cohort	7	2/5	45.29 (6.9)	Adults
Nardone et al. (2014) [33]	USA	Cohort	67	47/20	6	Children
Bian et al. (2024) [19]	China	Cohort	43	31/12	9.8 (1.9)	Children
Ramos and Gama (2017) [34]	Brazil	RCT	25	20/5	8.18 (2.03)	Children
Yılmazer et al. (2019) [23]	Turkey	RCT	10	10 females	30.4 (8.1)	Adults
Bequignon et al. (2013) [35]	France	Cohort	62	2/60	28.1	Adults
Valadez et al. (2012) [36]	Mexico	Cohort	40	30/10	8	Children

Treatment Strategies Used in VCN

Different treatment strategies for VCNs were reported in the included studies. Fourteen articles involved speech or voice therapy, nine included surgical interventions, three covered medical management with drugs, and one focused on observation therapy. Different outcomes were measured after therapy including Consensus Auditory-Perceptual Evaluation of Voice (CAPE-V), Pediatric Voice Handicap Index (pVHI), videolaryngostroboscopy (VLS), Voice Handicap Index (VHI), VCNs grade, Dysphonia Severity Index (DSI), maximum phonation time (MPT), acoustic parameters including jitter (%) and shimmer (%), functional score, physical score, and emotional score. Follow-up for measurement of the outcomes after treatment ranged from two weeks to five years (Table 2). The included studies aimed to investigate the effects of different management strategies on VCNs by the assessment of different outcomes before and after treatment (Table 3).

**Table 2:** Overview of different treatment strategies in vocal cord nodules and the assessed outcomes. VoxMetria (Lisle, IL: CTS, Inc.); Acublade scanner system (Illit, Israel: Lumenis)

Study ID	Treatment	Mechanism	Outcomes	Follow-up
Liu et al. (2024) [21]	Speech therapy, surgery	-	Consensus Auditory-Perceptual Evaluation of Voice (CAPE-V), Pediatric Voice Handicap Index (pVHI), Computerized voice assessment, dysphonia	1 year
Ma et al. (2024) [24]	Vocal hygiene education with resonant voice therapy	-	Auditory-perceptual evaluation of overall dysphonia severity, pVHI, and the Children’s Voice Handicap Index-10 (CVHI-10). Acoustic analysis of fundamental frequency, jitter, shimmer, and noise-to-harmonic ratio	1.5 months
Hseu et al. (2024) [25]	Virtual voice therapy	-	CAPE-V scores and pVHI scores	2 months
Martins et al. (2020) [20]	Microsurgery	-	Total improvement (disappearance of vocal symptoms and of the laryngeal lesions); partial improvement (partial improvement of symptoms and/or maintenance of lesions); no improvement (maintenance or worsening of the symptoms and/or persistence of the lesion	-
Itigi et al. (2022) [26]	Medical management, speech therapy or phonosurgery	Cautiously using speech therapy in conjunction with oral steroids, anti-reflux drugs, or other medications. In other cases, an Acublade scanner system and a 40-Watt Lumenis CO_2_ laser were used to perform microlaryngeal phonosurgery	Videolaryngostroboscopy (VLS) and Voice Handicap Index (VHI)	1 month
Palash et al. (2022) [27]	Speech therapy, microsurgery followed by speech therapy	Voice therapy began with vocal hygiene instruction, covering normal voice production, recognizing and minimizing vocal abuse, staying hydrated, effects of irritants, laryngopharyngeal reflux, and specific medications. The therapy methods varied based on the patient's phonatory behavior and included a program called Exercises for Vocal Function. This eight-week program involved weekly sessions, with patients performing exercises at home twice daily for 15 minutes, except on therapy days	VHI	2 months
Akbulut et al. (2016) [28]	Speech therapy	-	VHI	1 month
Liu et al. (2022) [22]	-	-	-	1 year
Hartnick et al. (2018) [29]	Direct or Adventures in Voice (AIV) and indirect or My Voice Adventure (MVA) voice therapy	In the direct therapy arm of AIV, eight weekly modules over up to 12 weeks focused on replacing harmful vocal patterns with new ones using resonance training and motor learning techniques. This included at-home practice to reinforce the new patterns. The indirect therapy arm, MVA, aimed to reduce behaviors causing dysphonia through six sessions over 8-12 weeks, using a vocal hygiene program with games and activities for school-aged children. Key topics included normal vocal function, care, education about the voice mechanism, recognizing and modifying harmful behaviors, and discussing desired versus undesired voice production	Pediatric Voice-Related Quality of Life (PVRQOL)	3 months
Bakat et al. (2014) [30]	Speech therapy, microlaryngoscopic surgery	-	VHI, laryngoscopy	6 months
Mobarsa et al. (2019) [31]	Microsurgery	-	Amplitude, mucosal wave distribution, glottic closure, and VHI	3 weeks, and 10 weeks
Muslih et al. (2019) [32]	Microsurgery	-	Praat voice, VHI	2 weeks
Nardone et al. (2014) [33]	No treatment or behavioral modification only, targeted voice therapy with or without the treatment of associated conditions (gastroesophageal reflux and allergic rhinitis), surgical intervention	-	Vocal cord nodules (VCNs) grade or size	Monthly
Bian et al.(2024) [19]	ABCLOVE exercise after the treatment of budesonide	The training regimen included four main activities: breathing training, bubble blowing, resonance, and throat swinging. In throat swinging, participants vocalized "ah-ah" while relaxing neck and shoulder muscles and swinging the thyroid cartilage. They counted to ten while inhaling through the nose and contracting abdominal muscles. The resonance exercise involved making a nasal "hmm" sound with normal breathing. Bubble blowing entailed exhaling through a straw in water to loosen vocal cords, followed by vocalization while inhaling through the nose and engaging abdominal muscles	Subjective voice assessment, dysphonia severity index (DSI), maximum phonation time (MPT), acoustic parameters including jitter (%) and shimmer (%), pVHI, functional score, physical score, and emotional score	3 months
Ramos and Gama (2017) [34]	Semi-Occluded vocal tract exercises (speech therapy)	In the control group, participants underwent complete voice rest, playing video games without phonating. Voice samples were recorded before and at the first, third, fifth, and seventh minutes of rest. The experimental group practiced straw phonation using a stiff plastic straw (8.7 cm long, 1.5 mm diameter), making noise while blowing through the straw positioned between the lips and teeth. Voice samples were recorded before and at the same intervals using VoxMetria software and a unidirectional condenser microphone. Participants vocalized prolonged vowels and counted from 1 to 10 at a comfortable loudness and pitch	Grade Roughness Breathiness Asthenia/Strain Instability scale. For acoustic analysis, fundamental frequency, jitter, shimmer, glottal to noise excitation ratio, and noise parameters	NR
Yılmazer et al. (2019) [23]	Speech therapy	-	VHI-10 questionnaire, MPT and s/z ratio, acoustic and aerodynamic analyses, and VLS examinations	2 months
Bequignon et al. (2013) [35]	Speech therapy, surgery	-	VHI	5 years
Valadez et al. (2012) [36]	Speech therapy	-	Shimmer, jitter, and fundamental frequency	5 months

**Table 3:** Summary of findings of the included studies. pVHI: pediatric voice handicap index; VCNs: vocal cord nodules; CAPE-V: Consensus Auditory-Perceptual Evaluation of Voice; VHI: Voice Handicap Index; MPT: maximum phonation time; DSI: dysphonia severity index; PVRQOL: Pediatric Voice-Related Quality of Life; VLS: videolaryngostroboscopy Acublade scanner system (Illit, Israel: Lumenis)

Study ID	Treatment	Mechanism	Outcomes	Follow-up
Liu et al. (2024) [21]	Speech therapy, surgery	-	Consensus Auditory-Perceptual Evaluation of Voice (CAPE-V), Pediatric Voice Handicap Index (pVHI), computerized voice assessment, dysphonia	1 year
Ma et al. (2021) [24]	Vocal hygiene education with resonant voice therapy	-	Auditory-perceptual evaluation of overall dysphonia severity, pVHI, and the Children’s Voice Handicap Index-10 (CVHI-10). Acoustic analysis of fundamental frequency, jitter, shimmer, and noise-to-harmonic ratio	1.5 months
Hseu et al. (2024) [25]	Virtual voice therapy	-	CAPE-V scores and pVHI scores	2 months
Martins et al. (2020) [20]	Microsurgery	-	Total improvement (disappearance of vocal symptoms and of the laryngeal lesions); partial improvement (partial improvement of symptoms and/or maintenance of lesions); no improvement (maintenance or worsening of the symptoms and/or persistence of the lesion	-
Itigi et al. (2022) [26]	Medical management, speech therapy or phonosurgery	Cautiously using speech therapy in conjunction with oral steroids, anti-reflux drugs, or other medications. In other cases, an Acublade scanner system and a 40-Watt Lumenis CO_2_ laser were used to perform microlaryngeal phonosurgery	Videolaryngostroboscopy (VLS) and Voice Handicap Index (VHI)	1 month
Palash et al. (2022) [27]	Speech therapy, microsurgery followed by speech therapy	Voice therapy began with vocal hygiene instruction, covering normal voice production, recognizing and minimizing vocal abuse, staying hydrated, effects of irritants, laryngopharyngeal reflux, and specific medications. The therapy methods varied based on the patient's phonatory behavior and included a program called Exercises for Vocal Function. This eight-week program involved weekly sessions, with patients performing exercises at home twice daily for 15 minutes, except on therapy days	VHI	2 months
Akbulut et al. (2016) [28]	Speech therapy	-	VHI	1 month
Liu et al. (2022) [22]	-	-	-	1 year
Hartnick et al. (2018) [29]	Direct or Adventures in Voice (AIV) and indirect or My Voice Adventure (MVA) voice therapy	In the direct therapy arm of AIV, eight weekly modules over up to 12 weeks focused on replacing harmful vocal patterns with new ones using resonance training and motor learning techniques. This included at-home practice to reinforce the new patterns. The indirect therapy arm, MVA, aimed to reduce behaviors causing dysphonia through six sessions over 8-12 weeks, using a vocal hygiene program with games and activities for school-aged children. Key topics included normal vocal function, care, education about the voice mechanism, recognizing and modifying harmful behaviors, and discussing desired versus undesired voice production	Pediatric Voice-Related Quality of Life (PVRQOL)	3 months
Bakat et al. (2014) [30]	Speech therapy, microlaryngoscopic surgery	-	VHI, laryngoscopy	6 months
Mobarsa et al. (2019) [31]	Microsurgery	-	Amplitude, mucosal wave distribution, glottic closure, and VHI	3 weeks, and 10 weeks
Muslih et al. (2019) [32]	Microsurgery	-	Praat voice, VHI	2 weeks
Nardone et al. (2014) [33]	No treatment or behavioral modification only, targeted voice therapy with or without the treatment of associated conditions (gastroesophageal reflux and allergic rhinitis), surgical intervention	-	Vocal cord nodules (VCNs) grade or size	Monthly
Bian et al. (2024) [19]	ABCLOVE exercise after the treatment of budesonide	The training regimen included four main activities as follows: breathing training, bubble blowing, resonance, and throat swinging. In throat swinging, participants vocalized "ah-ah" while relaxing neck and shoulder muscles and swinging the thyroid cartilage. They counted to ten while inhaling through the nose and contracting abdominal muscles. The resonance exercise involved making a nasal "hmm" sound with normal breathing. Bubble blowing entailed exhaling through a straw in water to loosen vocal cords, followed by vocalization while inhaling through the nose and engaging abdominal muscles	Subjective voice assessment, dysphonia severity index (DSI), maximum phonation time (MPT), acoustic parameters including jitter (%) and shimmer (%), pVHI, functional score, physical score, and emotional score	3 months
Ramos and Gama (2017) [34]	Semi-Occluded vocal tract exercises (speech therapy)	In the control group, participants underwent complete voice rest, playing video games without phonating. Voice samples were recorded before and at the first, third, fifth, and seventh minutes of rest. The experimental group practiced straw phonation using a stiff plastic straw (8.7 cm long, 1.5 mm diameter), making noise while blowing through the straw positioned between the lips and teeth. Voice samples were recorded before and at the same intervals using VoxMetria software and a unidirectional condenser microphone. Participants vocalized prolonged vowels and counted from 1 to 10 at a comfortable loudness and pitch	Grade Roughness Breathiness Asthenia/Strain Instability scale. For acoustic analysis, fundamental frequency, jitter, shimmer, glottal to noise excitation ratio, and noise parameters	NR
Yılmazer et al. (2019) [23]	Speech therapy	-	VHI-10 questionnaire, MPT and s/z ratio, acoustic and aerodynamic analyses, and VLS examinations	2 months
Bequignon et al. (2013) [35]	Speech therapy, surgery	-	VHI	5 years
Valadez et al. (2012) [36]	Speech therapy	-	Shimmer, jitter, and fundamental frequency	5 months

Voice/Speech Therapy

Voice or speech therapy was the main conservative treatment, reported in 14 of the 18 included articles. It involves various techniques such as virtual voice therapy, direct or Adventures in Voice (AIV), indirect or My Voice Adventure (MVA) therapy, and ABCLOVE exercise. Different methods were used for these types of voice therapy. Virtual voice therapy was conducted using telemedicine, where the doctor communicates with the patient over the Internet to facilitate continuous management and follow-up. Direct voice therapy (AIV) involves replacing pre-existing phonotraumatic vocal patterns with new ones established through motor learning techniques, such as resonance training, behavioral modeling, and shaping. MVA is based on a vocal hygiene program created by Nilson and Schneiderman and employs commonly used techniques for treating phonotraumatic behaviors in school-aged children [37]. Games and other activities are part of the treatment to raise awareness and alter phonotraumatic vocal patterns. The ABCLOVE exercise, performed after treatment with budesonide, consists of four parts as follows: breathing training, bubble blowing, resonance, and throat swinging (Table 2).

While some studies investigated different types of speech therapy with no comparisons, others compared different methods of speech therapy or compared speech therapy to other treatment strategies such as surgical interventions. Ma et al. showed that the use of vocal hygiene education with resonant voice therapy is associated with improvements in pVHI scores and overall dysphonia [24]. Virtual voice therapy showed significant improvements in quality of life and perceptual CAPE-V scores in overall symptom severity [25]. The ABCLOVE method by Bian et al. showed that for school-age children with VCNs, hoarseness and roughness ratings were significantly lowered with ABCLOVE therapy [19]. Additionally, the ABCLOVE therapy group experienced a substantial decline in functional, physical, emotional, and overall pVHI scores (Table 3).

In comparing different speech therapy approaches, Hartnick et al. examined direct (AIV) and indirect (MVA) speech therapies. They reported that children with VCNs benefited significantly from both approaches, with no significant differences observed between the two methods (Table 3) [29]. Some studies compared speech therapy with surgical interventions such as Liu et al., who showed that both treatments are associated with good outcomes [21]. However, speech therapy is the primary choice for its non-invasiveness, while surgery is rarely indicated due to its invasiveness, although it is associated with faster recovery. Palash et al. reported comparable efficacy between speech therapy and surgery, with no significant differences [27]. On the other hand, surgery may have better results in case of failure of voice therapy.

Interestingly, data by Bakat et al. suggest that the choice between conservative (voice therapy) and invasive (surgery) treatment may depend on the nodule characteristics. In the cohort studied by Bakat et al. (n=18), objective assessments using rigid fiber optic laryngoscopy (FOL) with a 70° endoscope allowed for the classification of VCNs based on their type, glottic closure pattern, and texture (i.e., hard or soft). The authors observed that all patients with soft nodules (15 out of 18) experienced significant improvements, both in objective and subjective voice outcomes following voice therapy. However, the few patients (three out of 18; 16.67%) with hard nodules did not show significant improvement after voice therapy but fully recovered after microlaryngoscopic surgery (Table 3).

Pharmacological Management

Pharmacological management was the least frequent treatment modality in the included studies, mentioned in only three studies. Itigi et al. showed that medical management with oral corticosteroids and anti-reflux agents was associated with improvements in VLS and VHI [26]. However, in all cases, pharmacological treatment was combined with phonosurgery, and some patients also underwent speech therapy. Therefore, the study’s design does not enable ascertaining that improvements in voice outcomes are related to medical management protocols. On the other hand, Nardone et al. combined speech therapy with pharmacological treatment of associated conditions (gastroesophageal reflux and allergic rhinitis) and observed improvements in VCN grade, while observation therapy showed no effectiveness [33]. Bian et al. reported that speech therapy using ABCLOVE methods after budesonide (corticosteroids) treatment was associated with better improvements in speech outcomes, as previously mentioned [19]. Therefore, pharmacological treatment cannot be used as a standalone measure in the management of VCNs, but as an adjunct treatment for the associated conditions (Table 3).

Surgical Management

Surgical management was reported in nine of the included studies, with surgical techniques varying among studies (Table 2). Microsurgical excision [20,27,30-32] and phonosurgery were carried out using Acublade scanner system (Illit, Israel: Lumenis) and a 40-watt Lumenis CO_2_ laser [26]. Phonosurgery, reported by Itigi et al., showed improvement in both VLS and VHI [26]. Similarly, Mobarsa et al. showed that microsurgery was associated with improved glottic closure, mucosal wave amplitude, and VHI (Table 3) [31].

As previously reported, some studies compared the outcomes of surgery and speech therapy. Liu et al. reported that conservative treatments including speech therapy and vocal hygiene continue to be the primary choice for patients with VCNs [21]. However, surgical excision of VCNs in selective patients is strongly associated with faster remission of dysphonic symptoms. Consistently, Bakat et al. showed that patients who were unresponsive to speech therapy showed full recovery after microlaryngoscopic surgery [30]. Consistently, Palash et al. and Nardone et al. showed no significant difference between the effect of surgery and voice therapy, while surgery produced faster responses [27,33]. Other studies showed contrasting results regarding surgery. Martins et al. reported that microsurgery for VCNs had a poor success rate, which makes speech therapy the most effective treatment as first-line therapy [20]. Surgery is further limited by the necessity for postoperative voice therapy. Bequignon et al. showed that lack of postoperative voice therapy was associated with a higher recurrence rate (56% of recurrent dysphonia without voice therapy versus 22% with voice therapy) (Table 3) [35].

Discussion

Summary of Findings

The current study provides insights into the management of VCNs in adults and children. Our findings demonstrated that different management strategies for VCNs exist, including voice therapy as the primary treatment strategy, surgery, and medical management of underlying conditions, in addition to observation management. All of the previously mentioned strategies were associated with improved voice performance, except for observation, which yielded minimal results.

While each strategy has its own advantages and disadvantages, voice therapy is the preferred option, especially for children, due to its non-invasive nature and lower risk of complications compared to surgical options. However, it takes longer to produce outcomes, often requiring many sessions over weeks to months. While surgery produces immediate improvements, it should be complemented with postoperative voice therapy to reduce the risk of recurrence and improve voice outcomes. Surgery appears to be more relevant in cases of failure of speech therapy or in the presence of hard nodules. Medical management was rarely assessed and is generally used as an adjunct to voice therapy for treating underlying conditions.

Pathophysiology of VCNs

Damage to the vocal cord tissues is the main cause of VCNs [38,39]. Initially, the nodule appears as edema in the vocal cord’s submucosa. The infiltration of blood and edema fluid into Reinke's space leads to the continued formation of the nodule. It progresses to hyalinized fibrous tissue depending on the type and degree of the causing factor. VCNs primarily modify the mass and consistency of the vocal cord due to subepithelial scar accumulation [40]. When nodules form, the membrane glottis may not close completely, leading to more turbulent air passing through the glottis. Attempts to produce voice can create a "vicious circle," exacerbating vocal injuries by increasing vocal fold collision forces, subglottal pressure, and muscular tension [41].

Anatomically, nodules can be classified into two groups based on their evolutive stage and laryngoscopic appearance as follows: soft or young nodules and hard or old nodules. Soft nodules are characterized by their acute nature and typically have a translucent, soft, and malleable texture, whereas hard or chronic nodules are stiff, white, and thick [42,43].

Mechanism of Action of Voice Therapy

Voice therapy is the preferred treatment method, where patients undergo re-education to learn correct voice usage through appropriate training programs. These programs, provided by a speech-language therapist, aim to educate patients to modify incorrect vocal habits and eliminate any misuse or abuse of their voice. If inappropriate voice habits are not addressed, there is a strong likelihood of recurrence. Voice hygiene instructions include minimizing or ceasing harmful vocal practices and administering targeted techniques and exercises to modify pitch, volume, or respiratory support for optimal vocalization [30].

From a physical perspective, voice hygiene techniques optimize aerodynamic forces (transglottal air pressure and glottal airflow) and acoustic properties (vocal loudness and pitch). This results in reduced muscle tension and hyperfunction, thereby decreasing damage to the vocal folds [44]. Compliance with voice hygiene is reported to decrease the size of most VCNS; however, the resolution of VCNs using voice therapy alone is debatable [23,45,46]. According to Yılmazer et al., only one of 10 VCNs disappeared following voice therapy, possibly because the nodule was soft [23]. It was noted that while hard nodules remained after voice therapy, soft nodules may resolve [47]. This may be related to hard nodules containing fibrous tissue in the subepithelial superficial lamina propria layer, which explains why hard nodules persist after voice therapy [23]. This probably explains why Bakat et al. found that surgical excision is necessary to eliminate hard nodules that are refractory to treatment by voice therapy alone [30].

Limitations of Each Treatment Approach

The lack of definite criteria for choosing the appropriate treatment modality - ranging from conservative approaches, such as educational or observational therapy, medications, and speech therapy, to invasive surgical options - highlights the need to understand each treatment method's limitations and preferred indications. Further analysis of the included studies indicates that each treatment modality is preferred in specific situations and limited by certain circumstances. Starting with conservative approaches, Ongkasuwan and Friedman recommended observation and follow-up, considering children's inadequate treatment compliance and their tendency for self-healing during adolescence [48]. In contrast, Nardone et al. found no effect of observation and follow-up compared to other treatments [33]. This may be due to the functional and anatomical impairments of the vocal cords and the presence of associated comorbidities, such as inflammatory conditions (rhinitis and sinusitis), which need adequate treatment.

Regarding medical management, Hooper and Levine et al. recommended initial treatment of any associated illnesses leading to the development of VCNs [5,49]. However, a purely pharmacological approach may lead to extended or repeated use of these treatments, which exposes patients to severe adverse effects such as antibiotic resistance (in the case of antibiotics), diabetes mellitus, and osteoporosis (in the case of corticosteroids) [50,51].

Consequently, voice hygiene or vocal training appears to be the most reasonable approach for most patients and is recommended by numerous professionals in recent times [36,52]. However, its efficacy may be delayed, and the resulting long-term dysphonia may negatively impact children's quality of life [53]. Nardone et al. observed that surgical excision resulted in a one-grade decrease in VCN size after 12.5 months, compared to 33.3 months for speech therapy [33]. Consistently, Liu et al. demonstrated better improvement of dysphonic disorders following surgical removal of VCNs, by reference to speech therapy [21]. This delay in efficacy of speech therapy results in increased financial burden and worsened children’s psychological condition. According to Mori and Choi and Cotton, non-surgical therapy, including speech therapy and other conservative approaches, can delay recovery for months or years, placing a significant financial and psychological strain on both parents and children [16,54]. Therefore, owing to rapid improvement by removing VCNs, surgical interventions reduce the psychological impact of long-term dysphonia and eliminate the costs associated with long-term psychological management and other indirect costs. However, symptom relief after surgery is temporary and should be complemented with voice therapy. Béquignon et al. showed that the lack of postoperative voice therapy was associated with a higher recurrence rate (56% of recurrent dysphonia without voice therapy versus 22% with voice therapy) [35]. Thus, surgical interventions alone are not adequate for managing VCNs.

Effects of Various Factors on Treatment Success

The present review identified several factors that are associated with treatment success and may influence the choice of treatment modality. Mori compared the efficacy of different treatment modalities in children with VCNs, using either patient or parent-reported symptomatic improvement or resolution, and analyzed the factors associated with treatment efficacy [16]. Surgery showed the best therapeutic results among prepubertal patients, while no significant differences were observed between observation, voice hygiene, and speech therapy. In contrast, postpubescent patients showed no discernible variation in response to treatment modalities, and nearly all of them improved. Nardone et al. found a greater rate of reduction in VCN size among postpubescent children compared to prepubertal ones, regardless of the treatment [33]. This was attributed to improved compliance with vocal hygiene and hormonal changes associated with puberty. Furthermore, the development of the vocal folds during adolescence may result in a shift in the position of the highest shear stress during phonation. This may lessen the trauma to nodules that have already formed, causing them to shrink and resolve [33].

Another factor that may influence treatment is the nodule morphological features. While data by Liu et al. and Nardone et al. suggest a correlation between the intensity of dysphonic symptoms and VCN size, this relationship is debatable [21,33]. According to Shah et al., voice quality and VCN size did not correlate significantly [55]. On the other hand, Nuss et al. showed a substantial relationship between nodule size and strain, pitch, loudness, roughness, and overall symptoms’ severity [56]. Further correlations between VCN morphological features and voice analysis indices have been examined in prior works [55,56]. A few studies, however, have analyzed morphological features as prognostic factors. Among these is the study by Liu et al., which explored the prognostic value of VCN morphological features in the absence of intervention [22]. They graded nodules based on size, contour, and maturity using static fiber-optic imaging of children's larynxes. The findings showed that continuous mechanical vibration and friction do not alter nodule growth but rather accelerate nodule maturation, indicated by its hard, pale, and well-defined shape. They found that large, mature nodules typically take longer to heal. Interestingly, the authors observed no relationship between VCN morphology and sinusitis or allergic rhinitis, indicating that VCN morphology is not significantly impacted by the local inflammatory state. Other prognostic factors included age and length of dysphonia, both of which showed a positive correlation with delayed recovery [22].

Proposed Algorithm for the Management of VCNs

**Figure 2:**
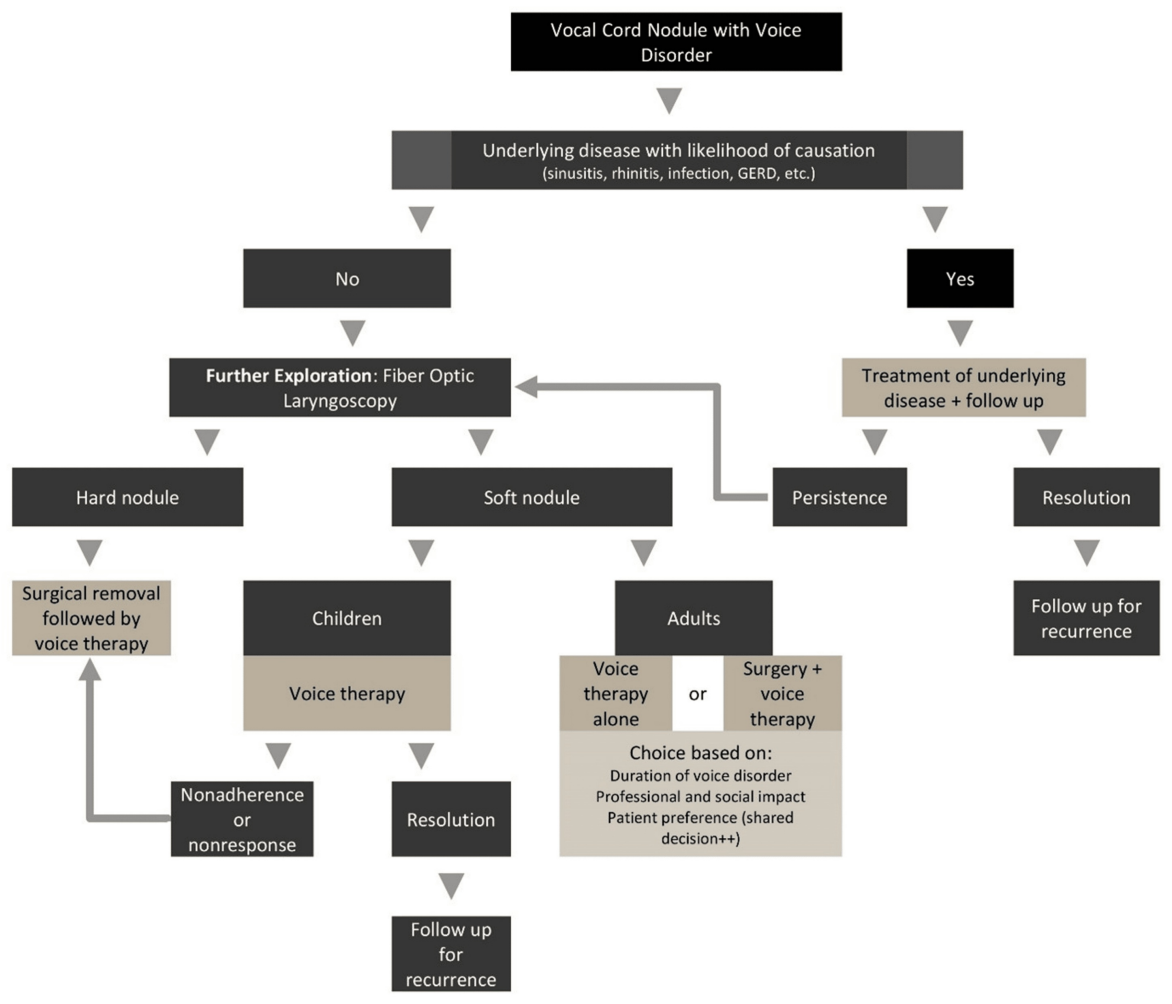
Management algorithm for vocal cord nodules with voice disorders. The image is created by the authors of this study using Microsoft PowerPoint 365.

Based on our findings, the choice of the management strategy, notably the first-line treatment, should consider certain key factors, specifically the existence of an underlying condition, the patient’s age, nodule maturity, and symptom duration. Consequently, the management algorithm begins with identifying any underlying disease likely contributing to the condition, such as upper airway inflammation of infection, GERD, etc. (Figure 2). If an underlying disease is present, it should be treated, and the patient should be followed up. If the disorder persists after treatment or if no underlying disease is identified, further exploration, notably through FOL, should be conducted to characterize the nodule morphological features. For hard nodules indicative of advanced disease, surgical removal followed by voice therapy is recommended given the likelihood of resistance to conservative treatment. For soft nodules, the first-line treatment depends on the patient’s age. In children, soft nodules are managed with voice therapy alone, while in adults, a choice between surgery plus voice therapy and voice therapy alone is made based on the duration of the voice disorder, professional and social impact of the voice handicap, and the patient preference. In both children and adults, non-adherence or non-response to voice therapy should prompt a discussion regarding surgical indications. In all cases, resolution should be closely monitored for recurrence.

Strengths and Limitations

This review offers a comprehensive assessment of treatment modalities for managing VCNs in adults and children. Furthermore, it analyzed the success and failure factors associated with each modality, thereby aiding in making appropriate treatment choices. The deep analysis of this data enabled the elaboration of a practical algorithm for the management of VCNs that considered the key factors. However, the study is limited by heterogeneity among the included articles, stemming from variations in patient characteristics, treatment types, study designs (comparative or non-comparative), assessment methods, mechanisms of action, and the diverse outcomes evaluated by the studies.

Future Directions

The major gap in the literature highlighted by this review is the lack of well-designed randomized controlled trials (RCTs). These RCTs should be age-specific and take into account the clinical and morphological characteristics of the treatment response identified in this review.

## Conclusions

This review provides insights into the management of VCNs in both adults and children, highlighting the various conservative and invasive treatment strategies available. While all these strategies can reduce nodule size and improve voice performance, voice therapy is particularly preferred, especially for children, due to its non-invasive nature. However, it requires a longer time to produce results. Surgery offers immediate improvement but should be complemented with postoperative voice therapy to reduce recurrence. Medical management is generally used as an adjunct to voice therapy for underlying conditions.

We observed significant heterogeneity among the included studies, impacting the consistency of the findings. Future research should focus on well-designed randomized controlled trials that are age-specific and take into account the clinical and morphological characteristics of treatment response identified in this review. This will help to establish more definitive guidelines for the management of VCNs and improve patient outcomes.

## References

[1] Pontes P, Kyrillos L, Behlau M, De Biase N, Pontes A (2002). Vocal nodules and laryngeal morphology. J Voice.

[2] Birchall MA, Carding P (2019). Vocal nodules management. Clin Otolaryngol.

[3] Gray SD, Smith ME, Schneider H (1996). Voice disorders in children. Pediatr Clin North Am.

[4] Choi SS, Zalzal GH (2015). Voice disorders. Cummings Pediatric Otolaryngology.

[5] Hooper CR (2004). Treatment of voice disorders in children. Lang Speech Hear Serv Sch.

[6] Morrison MD, Rammage LA (1993). Muscle misuse voice disorders: description and classification. Acta Otolaryngol.

[7] Hirschberg J, Dejonckere PH, Hirano M, Mori K, Schultz-Coulon HJ, Vrticka K (1995). Voice disorders in children. Int J Pediatr Otorhinolaryngol.

[8] Ruddy BH, Sapienza CM (2004). Treating voice disorders in the school-based setting: working within the framework of IDEA. Lang Speech Hear Serv Sch.

[9] Pedersen M, McGlashan J (2012). Surgical versus non-surgical interventions for vocal cord nodules. Cochrane Database Syst Rev.

[10] Watts CR (2012). Behavioral voice therapy in school-age children with vocal fold nodules. EBP Briefs.

[11] Simpson B, Rosen C (2008). Nonsurgical treatment of voice disorders. Operative Techniques in Laryngology.

[12] Gartner-Schmidt JL, Roth DF, Zullo TG, Rosen CA (2013). Quantifying component parts of indirect and direct voice therapy related to different voice disorders. J Voice.

[13] Vashani K, Murugesh M, Hattiangadi G (2010). Effectiveness of voice therapy in reflux-related voice disorders. Dis Esophagus.

[14] De Bodt MS, Ketelslagers K, Peeters T (2007). Evolution of vocal fold nodules from childhood to adolescence. J Voice.

[15] Mornet E, Coulombeau B, Fayoux P, Marie JP, Nicollas R, Robert-Rochet D, Marianowski R (2014). Assessment of chronic childhood dysphonia. Eur Ann Otorhinolaryngol Head Neck Dis.

[16] Mori K (1999). Vocal fold nodules in children: preferable therapy. Int J Pediatr Otorhinolaryngol.

[17] Eapen BR (2006). EndNote 7.0. Indian J Dermatol Venereol Leprol.

[18] (2024). Rayyan: faster systematic literature reviews. https://rayyan.ai/.

[19] Bian Y, Wang J, Zhang H, Yin X, Zhang Y (2024). Voice treatment of school-aged children with vocal nodules with ABCLOVE rehabilitation. Pediatr Neonatol.

[20] Martins RH, Siqueira DB, Dias NH, Gramuglia AC (2020). Laryngeal microsurgery for the treatment of vocal nodules and cysts in dysphonic children. Folia Phoniatr Logop.

[21] Liu K, Mousset M, Schafer A (2024). Surgical management of vocal cord nodules in children: trends and outcomes. Am J Otolaryngol.

[22] Liu J, Cao W, Sun DH, Wu L, Sun J, Xu B, Fu Y (2022). Vocal nodules in children: laryngoscopic morphological classification aids prognostic judgment. Front Pediatr.

[23] Yılmazer R, Süzer GK, Süoğlu Y (2019). The efficacy of voice therapy in vocal cord nodules. Tr-ENT.

[24] Ma EP, Cheung YC, Siu AK, Lo JF (2024). The effectiveness of vocal hygiene education with resonant voice therapy for school-aged children with vocal nodules. J Voice.

[25] Hseu AF, Spencer G, Jo S, Kagan S, Thompson K, Woodnorth G, Nuss RC (2024). Telehealth for treatment of pediatric dysphonia. J Voice.

[26] Itigi S, Sikdar A, Phatak S, Nivsarkar S (2022). Comparative study of videolaryngostroboscopic findings and voice handicap index before and after treatment in patients presenting with hoarseness due to non malignant lesions. Indian J Otolaryngol Head Neck Surg.

[27] Palash CS, Uddin MM, Salam KS, Ahmad SM, Sayied MJ, Haque MH (2022). Surgical and non-surgical treatment outcome assessed by voice handicap index of patients with vocal fold nodule. Bangladesh J Otorhinolaryngol.

[28] Akbulut S, Gartner-Schmidt JL, Gillespie AI, Young VN, Smith LJ, Rosen CA (2016). Voice outcomes following treatment of benign midmembranous vocal fold lesions using a nomenclature paradigm. Laryngoscope.

[29] Hartnick C, Ballif C, De Guzman V (2018). Indirect vs direct voice therapy for children with vocal nodules: a randomized clinical trial. JAMA Otolaryngol Head Neck Surg.

[30] Bakat B, Roy A, Raychaudhuri BK (2014). Does voice therapy cure all vocal fold nodules?. Int J Phonosurg Laryngol.

[31] Mobarsa V, Samdani SK, Gurjar VS (2019). Outcome analysis of microlaryngeal surgery for benign lesions of vocal cord using videostroboscopy and voice handicap index. Indian J Otolaryngol Head Neck Surg.

[32] Muslih I, Herawati S, Pawarti DR (2019). Association between voice handicap index and praat voice analysis in patients with benign vocal cord lesion before and after microscopic laryngeal surgery. Indian J Otolaryngol Head Neck Surg.

[33] Nardone HC, Recko T, Huang L, Nuss RC (2014). A retrospective review of the progression of pediatric vocal fold nodules. JAMA Otolaryngol Head Neck Surg.

[34] Ramos LA, Gama AC (2017). Effect of performance time of the semi-occluded vocal tract exercises in dysphonic children. J Voice.

[35] Béquignon E, Bach C, Fugain C, Guilleré L, Blumen M, Chabolle F, Wagner I (2013). Long-term results of surgical treatment of vocal fold nodules. Laryngoscope.

[36] Valadez V, Ysunza A, Ocharan-Hernandez E, Garrido-Bustamante N, Sanchez-Valerio A, Pamplona MC (2012). Voice parameters and videonasolaryngoscopy in children with vocal nodules: a longitudinal study, before and after voice therapy. Int J Pediatr Otorhinolaryngol.

[37] Nilson H, Schneiderman CR (1983). Classroom program for the prevention of vocal abuse and hoarseness in elementary school children. Lang Speech Hear Serv Sch.

[38] Hirano M, Kurita S, Matsuo K, Nagata K (1980). Laryngeal tissue reaction to stress. Transcripts of the Ninth Symposium: Care of the Professional Voice.

[39] Titze IR (1994). Mechanical stress in phonation. J Voice.

[40] Franco RA, Andrus JG (2007). Common diagnoses and treatments in professional voice users. Otolaryngol Clin North Am.

[41] Hillman RE, Holmberg EB, Perkell JS, Walsh M, Vaughan C (1989). Objective assessment of vocal hyperfunction: an experimental framework and initial results. J Speech Hear Res.

[42] Sataloff RT, Spiegel JR, Carroll LM, Schiebel BR, Darby KS, Rulnick R (1988). Strobovideolaryngoscopy in professional voice users: results and clinical value. J Voice.

[43] Sataloff RT, Spiegel JR, Hawkshaw MJ (1991). Strobovideolaryngoscopy: results and clinical value. Ann Otol Rhinol Laryngol.

[44] Holmberg EB, Hillman RE, Perkell JS (1988). Glottal airflow and transglottal air pressure measurements for male and female speakers in soft, normal, and loud voice. J Acoust Soc Am.

[45] Lu D, Chen F, Yang H (2018). Changes after voice therapy in acoustic voice analysis of Chinese patients with voice disorders. J Voice.

[46] Holmberg EB, Hillman RE, Hammarberg B, Södersten M, Doyle P (2001). Efficacy of a behaviorally based voice therapy protocol for vocal nodules. J Voice.

[47] Chernobelsky SI (2007). The treatment and results of voice therapy amongst professional classical singers with vocal fold nodules. Logoped Phoniatr Vocol.

[48] Ongkasuwan J, Friedman EM (2013). Is voice therapy effective in the management of vocal fold nodules in children?. Laryngoscope.

[49] Levine BA, Jacobs IN, Wetmore RF, Handler SD (1995). Vocal cord injection in children with unilateral vocal cord paralysis. Arch Otolaryngol Head Neck Surg.

[50] Kiss C, Connoley D, Connelly K, Horne K, Korman T, Woolley I, Lau JS (2022). Long-term outcomes in patients on life-long antibiotics: a five-year cohort study. Antibiotics.

[51] Sendrasoa FA, Ranaivo IM, Raherivelo AJ, Rapelanoro Rabenja F, Ramarozatovo LS (2021). Adverse effects of long-term oral corticosteroids in the department of dermatology, Antananarivo, Madagascar. Clin Cosmet Investig Dermatol.

[52] Rodríguez-Parra MJ, Adrián JA, Casado JC (2011). Comparing voice-therapy and vocal-hygiene treatments in dysphonia using a limited multidimensional evaluation protocol. J Commun Disord.

[53] Connor NP, Cohen SB, Theis SM, Thibeault SL, Heatley DG, Bless DM (2008). Attitudes of children with dysphonia. J Voice.

[54] Choi SS, Cotton RT (1989). Surgical management of voice disorders. Pediatr Clin North Am.

[55] Shah RK, Engel SH, Choi SS (2008). Relationship between voice quality and vocal nodule size. Otolaryngol Head Neck Surg.

[56] Nuss RC, Ward J, Huang L, Volk M, Woodnorth GH (2010). Correlation of vocal fold nodule size in children and perceptual assessment of voice quality. Ann Otol Rhinol Laryngol.

